# Parental Psychiatric Symptoms and Child Emotional and Behavioural Difficulties: Exploring the Mediating Role of Parental Reflective Functioning in Parents With Mental Disorders

**DOI:** 10.1111/eip.70208

**Published:** 2026-06-25

**Authors:** Amanda Kaas, Olivia Pepin, Sebastian Simonsen, Per Sørensen, Mette Væver, Ida Egmose, Emilie Hestbaek

**Affiliations:** ^1^ Department of Psychology University of Copenhagen Copenhagen Denmark; ^2^ Mental Health Center Stolpegaard Copenhagen University Hospital Herlev and Gentofte Denmark; ^3^ National Institute of Public Health University of Southern Denmark Copenhagen Denmark

**Keywords:** behavioural problems, child development, emotional problems, mentalization, parental reflective functioning, parenting, psychopathology

## Abstract

**Introduction:**

Children of parents with a mental disorder are at increased risk of developing mental health problems. A potentially important construct in the intergenerational transmission of mental health problems is parental mentalizing, referred to as parental reflective functioning (PRF). The aim of this study was to examine the links between parental psychiatric symptom severity, PRF, and child emotional and behavioural difficulties among parents with a mental disorder. Furthermore, we sought to explore the potential mediating role of PRF.

**Methods:**

The study involved parents with various diagnosed mental disorders (*N* = 70) with a child aged 0–17. Psychiatric symptom severity was assessed using the Brief Symptom Inventory, PRF was assessed with the Parental Reflective Functioning Questionnaire, and child emotional and behavioural difficulties were assessed with the Strengths and Difficulties Questionnaire. Linear regressions and Hayes PROCESS macro were utilised to examine the links between the variables and test for the mediating role of PRF.

**Results:**

Parental psychiatric symptom severity was significantly related to higher levels of child emotional and behavioural difficulties. Parental psychiatric symptom severity was closely associated with maladaptive PRF, as indicated by higher levels of pre‐mentalizing, which in turn was related to child emotional and behavioural difficulties. PRF did not mediate the relationship between parental psychiatric symptom severity and child emotional and behavioural difficulties.

**Conclusion:**

Parental psychiatric symptom severity and PRF are important for child emotional and behavioural difficulties. However, the relationship between parental psychiatric symptom severity and child emotional and behavioural difficulties was not mediated by PRF.

## 1 Introduction

Between 11% and 39% of children have at least one parent with a mental disorder (Abel et al. 2019; Bassani et al. 2009; Christesen et al. 2022; Maybery et al. 2009; Pierce et al. 2020). It is well established that children of parents with a mental disorder are at elevated risk of various adverse outcomes including school dropout, criminal offences, and somatic illness, resulting in a high use of healthcare and social services (Farahati et al. 2003; Heuckendorff 2021; Mok et al. 2016; Ranning et al. 2020). Of major concern is the consistent finding that these children are at heightened risk of developing a mental disorder themselves, referred to as the intergenerational transmission of psychopathology (Argent et al. 2020; Leijdesdorff et al. 2017). This transmission has primarily been investigated among single‐diagnostic samples (Uher et al. 2023), not accounting for the high degree of comorbidity between psychiatric diagnoses (Plana‐Ripoll et al. 2019). The transmission of psychopathology risk from parent to child may be attributed to a multitude of genetic and environmental mechanisms (Hosman et al. 2009; Reupert et al. 2013). Of particular importance for the psychological development of children are factors related to parenting, among which parental mentalizing, often operationalised as parental reflective functioning (PRF), has been highlighted as a central construct and is receiving increasing attention (Camoirano 2017). PRF is defined as the parent's capacity to reflect upon their own as well as their child's mental states, such as thoughts, feelings, and intentions (Slade 2005). Impaired PRF has been linked to the severity of psychiatric symptoms among clinical groups of parents (Håkansson et al. 2019; Krink and Ramsauer 2021) and early child emotional and behavioural difficulties (Camoirano 2017). However, only a few studies have examined the relationships between these three constructs altogether (Arikan and Kumru 2021; Keleynikov et al. 2024; Khoshroo and Mousavi 2022; Raouna et al. 2025). To our knowledge, none have done so in a clinical sample of parents with diagnosed mental disorders. It is important to further explore the role of PRF in the relationship between parental psychopathology and child emotional and behavioural difficulties, as this could lead to development of more targeted treatment supporting parenting in parents with mental disorders.

### 1.1 The Intergenerational Transmission of Psychopathology

Meta‐analyses show that children of parents with mental disorders across the diagnostic spectrum have a 1.5–3 times higher risk of developing a mental disorder compared to children of parents without a mental disorder (Rasic et al. 2014; Uher et al. 2023). Studies suggest that the severity of parental psychopathology is associated with increased risk of offspring psychopathology (Elbracht et al. 2023; Hammen and Brennan 2003). Signs of offspring vulnerability associated with parental psychopathology can already be traced in early childhood in the form of emotional and behavioural difficulties (Dean et al. 2018; Ivanova et al. 2022; Watkeys et al. 2022). Emotional and behavioural difficulties is an umbrella term for reactions discordant to the developmental stage of the child, impairing daily functioning (Poulou 2015). Emotional difficulties are typically characterised by fearfulness, sadness or social withdrawal, while behavioural difficulties often manifest as oppositional, aggressive or hyperactive behaviour (Cooper 1999). Although not clinical per se, these subdiagnostic difficulties are associated with risk for psychopathology later in life and are thus considered early risk markers of mental health problems (Hofstra et al. 2002; Roza et al. 2003). When screening for risk of psychopathology among children, it is important to consider both emotional and behavioural difficulties, as these often co‐exist (Achenbach et al. 2016; Gilliom and Shaw 2004). This is also underscored by studies showing that children often develop mental disorders dissimilar to their parents, indicating that psychopathology may be transferred from parent to child in a nonspecific manner (Uher et al. 2023). Thus, increased attention to a broad spectrum of difficulties among children of parents with mental disorders is crucial for early identification of early mental health problems.

### 1.2 Parental Reflective Functioning

The concept of PRF is rooted in the mentalizing framework and is a relationship‐specific capacity distinct from general mentalizing that involves the parent's understanding of their child's and their own state of mind (Slade 2005). Adaptive PRF is characterised by the parent showing genuine interest in the child's inner world and making meaningful interpretations of the child's behaviour, providing the basis for attending to the child's needs (Luyten, Nijssens, et al. 2017; Slade 2005). Empirically, adaptive PRF has been associated with various positive parenting behaviours, whilst maladaptive PRF has been associated with parental insensitivity and intrusive parenting (Ensink et al. 2019; Huth‐Bocks et al. 2014; Stuhrmann et al. 2022). PRF encompasses both trait‐ and state‐like features. Although it is primarily formed through early interactions with caregivers, elevated psychological distress is particularly linked to loss of controlled mentalizing. In more severe cases, this can lead to maladaptive PRF (Luyten, Nijssens, et al. 2017). Furthermore, maladaptive PRF has been linked to adverse child outcomes such as insecure child attachment (Zeegers et al. 2017), reduced child mentalizing (Ensink et al. 2016), and more recently child emotional and behavioural difficulties (e.g., Charpentier Mora et al. 2022; Condon et al. 2019; Dieleman et al. 2020; Khoshroo and Mousavi 2022). Studies have found PRF to be impaired among parents with mental disorders, including affective disorders, borderline personality disorder, and PTSD (Georg et al. 2023; Hestbaek et al. 2024; Moser et al. 2019; Zitzmann et al. 2025).

Taken together, empirical evidence has linked maladaptive PRF with parental psychopathology and early signs of child mental health problems. This could indicate that parental psychopathology may lead to a diminished capacity for reflection upon their child's mental states, which in turn may explain the adverse developmental outcomes often observed among children of parents with mental disorders. Therefore, it is important to empirically investigate whether PRF plays a mediating role in the relationship between parental psychiatric symptom severity and children's emotional and behavioural difficulties. A few studies have examined PRF as a mediator in the relationship between parental depressive symptoms and child emotional difficulties in community samples, finding mixed results (Keleynikov et al. 2024; Raouna et al. 2025).

The aim of this study was to investigate the links between parental psychiatric symptom severity, PRF, and child emotional and behavioural difficulties in a sample of parents with a diagnosed mental disorder. Furthermore, we explored the mediating role of PRF in the relationship between parental psychiatric symptom severity and child emotional and behavioural difficulties. Exploring these mechanisms in a clinical parent population is particularly important to prevent the intergenerational transmission of psychopathology. Informed by existing research, we hypothesised that:

1. Parental psychiatric symptom severity will be positively associated with child emotional and behavioural difficulties.
2. Parental psychiatric symptom severity will be positively associated with maladaptive PRF (pre‐mentalizing)
3. Maladaptive PRF (pre‐mentalizing) will be positively associated with child emotional and behavioural difficulties.
4. Maladaptive PRF (pre‐mentalizing) will mediate the relationship between parental psychiatric symptom severity and child emotional and behavioural difficulties.

## 2 Methods

### 2.1 Procedure and Participants

This study used a cross‐sectional design and is based on baseline data from a larger ongoing trial, the LIGHTHOUSE‐RCT (ID: NCT06315114), investigating the effects of a transdiagnostic mentalization‐based parenting program in parents with a mental disorder. After providing contact consent, parents were contacted with an invitation to participate in a program offered as an add‐on to their existing outpatient treatment. This recruitment strategy may have overrepresented parents experiencing parenting‐related difficulties and underrepresented those unable to engage in additional treatment. The trial is carried out at Psychotherapeutic Centre Stolpegaard and has been ethically approved by *the Regional Committee of Health Research Ethics (ID: H‐23064491).* Written and verbal informed consent was retrieved from all participants prior to data collection. The baseline data used in the current study were collected prior to randomisation during a 2‐h long session, where participants completed questionnaires and clinical interviews. Thus, all variables in this study were reported by parents. If parents had multiple children, they were instructed to report on the child causing them the greatest parenting distress. The eligibility criteria for the outpatient clinic and *the LIGHTHOUSE‐RCT* are presented in Table 1.

**TABLE 1:** Eligibility criteria for the outpatient clinic and the LIGHTHOUSE‐RCT.

	Inclusion criteria	Exclusion criteria
Clinic	Age > 18 years Seeking treatment for one of the following non‐psychotic disorders according to ICD‐10: obsessive‐compulsive disorder and anxiety disorders (F40‐42), posttraumatic stress disorder (DF43), personality disorders (F60‐61), or one of the previous disorders and a drug‐or alcohol related disorder (F10‐19) (i.e., ‘dual diagnosis disorder’)	Possibility of a learning disability (IQ < 75) A diagnosis of schizotypal personality disorder or antisocial personality disorder Presence of a comorbid psychiatric disorder that requires specialist treatment elsewhere Concurrent psychotherapeutic treatment outside the clinic
Trial	Parent of at least one child aged 0–17 years of age at baseline Fluent i.e., sufficient Danish language skills Parent is living with child or in regular contact Written informed consent	Acute suicidal risk or state of crises Lack of informed consent Acute child endangerment Participation in another parenting focused intervention simultaneously

### 2.2 Measures

#### 2.2.1 Parental Psychiatric Symptom Severity

Parental psychiatric symptom severity was assessed with the Danish version of the Brief Symptom Inventory (BSI; Derogatis and Melisaratos 1983), which is a 53‐item self‐report instrument covering level of distress on nine symptom domains. Participants rated their level of distress during the last 7 days on a 5‐point Likert scale ranging from 0 (not at all) to 4 (extremely). The Global Severity Index (GSI) is computed as the mean of all answered items, with allowance for up to 13 missing items in accordance with the manual. The GSI has shown high test–retest reliability (Derogatis and Melisaratos 1983). In the current study, Cronbach's Alpha for the GSI was 0.93, indicating excellent internal consistency.

#### 2.2.2 Parental Reflective Functioning

PRF was measured using the Danish version (Væver and Smith‐Nielsen 2005) of the Parental Reflective Functioning Questionnaire (PRFQ; Luyten, Mayes, et al. 2017). The PRFQ is a brief self‐report instrument consisting of 18 items, scored on a 7‐point Likert scale ranging from 1 (strongly disagree) to 7 (strongly agree) (Luyten, Mayes, et al. 2017). The PRFQ was originally validated for parents of children aged 0–5 years (Luyten, Mayes, et al. 2017). Subsequent studies have supported its use in samples including children up to 12 years (De Roo et al. 2019; Pazzagli et al. 2018), and its adolescent version (PRFQ‐A), involving only minor wording adaptations, has been validated among parents of adolescents up to 18 years (Park and Song 2024). In the current study, the original PRFQ was administered across the entire sample. Three subscales capture distinct features of PRF: (a) Pre‐Mentalizing (PM) reflecting non‐mentalizing modes of thinking (e.g., ‘My child cries around strangers to embarrass me’), (b) Certainty about Mental States (CMS), and (c) Interest and Curiosity (IC) in mental states (Luyten, Mayes, et al. 2017). Higher scores on the PM subscale reflect more maladaptive PRF, whilst medium levels on the CMS and IC subscales are indicative of optimal PRF (Luyten, Mayes, et al. 2017; Luyten, Nijssens, et al. 2017). Pre‐mentalizing was selected as our variable of interest as it represents severe disturbances in PRF, specifically capturing difficulties in comprehending the child's inner world and misattributions of their intentions as hostile. Although high and low scores on the CMS and IC subscales may capture maladaptive PRF, the non‐linear distribution of adaptive scores on these subscales were not suitable due to the nature of our analyses. The internal consistency, test–retest reliability and three‐factor structure of the PRFQ has been supported across samples of varying clinical risk (Carlone et al. 2023; Edler et al. 2023). The PM subscale had a Cronbach's Alpha of 0.67, indicating acceptable internal consistency.

#### 2.2.3 Child Emotional and Behavioural Difficulties

Child emotional and behavioural difficulties were measured using the Danish version (Obel et al. 2003) of the Strengths and Difficulties Questionnaire—Parent reported (SDQ; Goodman 1997). The SDQ consists of 25 items, which parents rated as either not true (0), true (1) or certainly true (2) (Goodman 1997). Four subscales (Emotional Symptoms, Conduct Problems, Hyperactivity and Peer Problems) can be summed to a Total Difficulties (TD) score. Two different versions were used for children above and below the age of 4, with age‐appropriate variations on items 18 and 22. There is support for the screening efficiency of the SDQ in terms of identifying clinical cases (Kovacs and Sharp 2014; Vugteveen et al. 2021; Warnick et al. 2008). In the current study, Cronbach's Alpha for the Total Difficulties scale was 0.83, indicating good internal consistency.

### 2.3 Statistical Analysis

**FIGURE 1:**
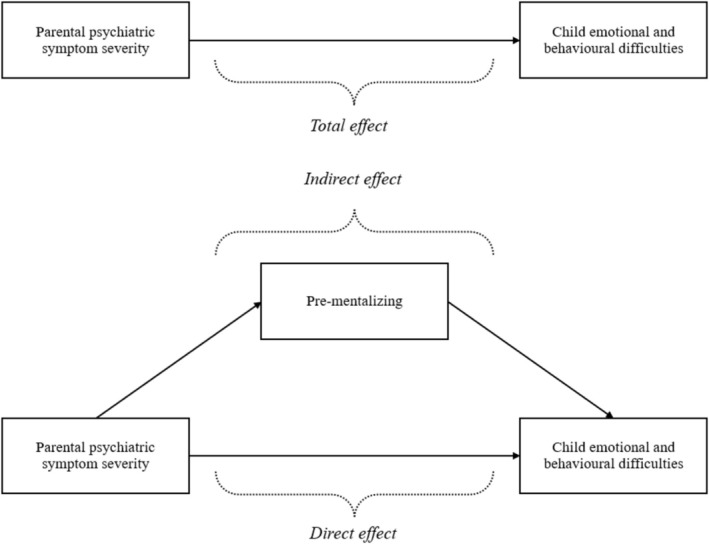
Conceptual diagram of the total, indirect and direct effect in our mediation model.

The statistical analyses were carried out in IBM Corp (2023) SPSS Statistics (Version 29.0.2). Across our main study variables (BSI‐GSI, PRFQ‐PM, SDQ‐TD), 4.2% of data was missing at item‐level. Further inspections hereof revealed that few respondents had the same missingness pattern and no items had more than five missing data points. For each scale (BSI‐GSI, PRFQ‐PM, SDQ‐TD) we created a binary missingness variable and conducted binary logistic regressions, which showed that missingness on each scale was not predicted by observed data on the other scales nor by sociodemographic variables (presented in Table 1). This was consistent with non‐significant results of a Little's MCAR‐test suggesting that the assumption of data being missing completely at random could not be rejected. Based on the low amount of missing data and the indications that missingness did not appear to be systematic, we decided to employ listwise deletion. For each analysis, listwise deletion was applied, excluding participants with missing values on variables included in that analysis. Participants with fewer than 13 missing items on the BSI‐GSI were retained (see Section 2.2.1). Next, we conducted visual inspections and Kolmogorov–Smirnov tests to ensure normality. The Pre‐Mentalizing scale on the PRFQ was found to be non‐normally distributed with a positive skew and a high peak. Because of this non‐normal distribution, we continued with Spearman's correlations. To examine for potential covariates, we conducted an exploratory correlation analysis between the study variables (BSI‐GSI, PRFQ‐PM, SDQ‐TD) and sociodemographic variables. As expected, this revealed a positive association between the SDQ Total Difficulties score and whether the child had a psychiatric diagnosis or not as reported by their parent (𝜌 = 0.26, *p* = 0.044). In the remainder of our analyses, we included child mental disorder as a covariate when child difficulties was the outcome, allowing us to estimate the effect of parental psychiatric symptom severity and pre‐mentalizing on child difficulties independent of child diagnostic status. Although the inclusion of child mental disorder as a covariate reduced the sample size in certain analyses due to six missing cases, it was retained to ensure methodological rigour and maintain the theoretical integrity of the analytic model. We tested our hypotheses with a series of analyses. First, we conducted a multiple linear regression with parental symptom severity (continuous variable) as a predictor, child difficulties (continuous variable) as the outcome, and child mental disorder (binary variable) as the covariate (H1). Secondly, we ran a simple linear regression with pre‐mentalizing (continuous) as a predictor and child difficulties (continuous) as the outcome (H2). Thirdly, we ran a multiple linear regression with pre‐mentalizing (continuous) as a predictor, child difficulties (continuous) as the outcome, and child mental disorder (binary) as a covariate (H3). Initially, we examined if the assumptions for linear regression analysis were met, assessing linearity, homoscedasticity, multicollinearity, normality and independence of residuals using scatterplots, P–P plots, VIF and Durbin‐Watson statistics. We found no evidence suggesting violations of the assumptions. To test our fourth and final hypothesis, we used Hayes' (2022) PROCESS macro version 4 for SPSS to determine whether pre‐mentalizing (M) mediated the relationship between parental symptom severity (X) and child emotional and behavioural difficulties (Y). We estimated different pathways linking X to Y in the mediation model (Figure 1). We applied 95% percentile bootstrap confidence intervals for the indirect effect (i.e., the mediation) based on 5000 samples. Statistical significance was determined at *p* < 0.05 for all analyses.

## 3 Results

### 3.1 Participant Characteristics

The study sample consisted of 70 parents. Sociodemographic characteristics of the sample are presented in Table 2. A majority of the parents were mothers (Table 2), and 38.6% of the sample presented with a primary diagnosis of PTSD (F43.1), 34.3% with emotionally unstable personality disorder (F60.3), and 15.7% with anxious [avoidant] personality disorder (F60.6) as classified in ICD‐10 (World Health Organization 2019). The remaining participants were seeking treatment for anxiety disorders (F41; 5.7%), obsessive‐compulsive disorder (F42; 4.3%), or alcohol‐related disorders (F10; 1.4%). In terms of comorbidity, 60.0% of the total sample had a secondary diagnosis, and 20.0% also had a third diagnosis. With regards to childhood adversity, 48.6% (*n* = 34) reported that they as a child experienced that one or both of their parents had a mental disorder, a drug‐related disorder, or attempted suicide. Moreover, 15.7% reported to have been placed out of home during their childhood. Despite these high‐risk characteristics, the participants had a relatively high educational level (Table 2). Furthermore, a majority reported that their child did not have a mental disorder at baseline (Table 2).

**TABLE 2:** Sociodemographic characteristics of the sample.

Variable		*n* (%)	*M* (SD)	Range
Parent gender	Female	59 (84.3)		
	Male	11 (15.7)		
Parent age			38.51 (7.86)	24–58
Child gender	Female	33 (47.1)		
	Male	35 (50.0)		
	Missing	2 (2.9)		
Child age			7.13 (4.41)	0–17
Parent educational level^a^	Low	11 (15.7)		
	Intermediate	24 (34.3)		
	High	35 (50.0)		
Occupation	Employed	16 (22.9)		
	Unemployed	41 (58.6)		
	Student	6 (8.6)		
	Other^b^	7 (10.0)		
Relationship status	Married	23 (32.9)		
	Living with partner	24 (34.3)		
	In a relationship	5 (7.1)		
	Single	18 (25.7)		
Child mental disorder	Yes	11 (15.7)		
	No	53 (75.7)		
	Missing	6 (8.6)		

*Note:* *N* = 70.

^a^ Definitions: Low educational level = Primary school or incomplete primary education; Intermediate educational level = Vocational education, other professional education, or upper secondary school; High educational level = Short, intermediate, or long higher education.

^b^ Other included stay‐at‐home, retired, and not further specified.

### 3.2 Correlational Analyses Between Parental Psychiatric Symptom Severity, Pre‐Mentalizing and Child Emotional and Behavioural Difficulties

Table 3 displays the descriptive statistics and zero‐order correlations between the main study variables. The parents' average scores on the BSI GSI (*M* = 1.24) were substantially higher than the mean of the general Danish population (*M* = 0.41) (Jensen 2025), as expected. When converted into a *T* score (*T* = 68.9) based on Danish norms, the participants' average GSI score was higher than the suggested clinical cutoff of *T* = 63 (Derogatis 1993), consistent with their clinical status. The children's average SDQ Total Difficulties score (*M* = 12.83) was on the verge between ‘close to average’ and ‘slightly raised’ according to the Danish norms for children aged 2–17 years (Arnfred et al. 2019). To date, no cut‐off scores or norms for the PRFQ exist. However, the parents' average scores were in the lower range on the Pre‐Mentalizing subscale (*M* = 2.14) (range = 1–7).

**TABLE 3:** Descriptive statistics and intercorrelations among study variables.

Variable	*M*	SD	1. BSI GSI	2. SDQ TD
1. BSI GSI	1.24	0.52	—	
2. SDQ TD	12.83	6.60	0.42***,^a^	—
3. PRFQ PM	2.14	0.94	0.55***,^b^	0.38**,^c^

Abbreviations: BSI GSI = Brief Symptom Inventory Global Severity Index (range 0–53); PRFQ PM = Parental Reflective Functioning Questionnaire Pre‐Mentalizing (range 1–7); SDQ TD = Strength and Difficulties Questionnaire Total Difficulties (range 0–40).

^a^ *N* = 61.

^b^ *N* = 62.

^c^ *N* = 61.

* *p* < 0.05.

** *p* < 0.01.

*** *p* < 0.001.

Spearman's correlations showed that parental psychiatric symptom severity was moderately associated with child emotional and behavioural difficulties (𝜌 = 0.42, *p* < 0.001) and strongly associated with pre‐mentalizing (𝜌 = 0.55, *p* < 0.001). A moderate correlation between pre‐mentalizing and child emotional and behavioural difficulties was found (𝜌 = 0.38, *p* = 0.003).

### 3.3 Parental Psychiatric Symptom Severity and Child Emotional and Behavioural Difficulties

The results of the three linear regression models are presented in Table 4. In line with our first hypothesis, a multiple linear regression found higher levels of parental psychiatric symptom severity (predictor) to be significantly associated with more child emotional and behavioural difficulties (outcome), when child mental disorder was entered as a covariate. The total model accounted for 25% of the variation in child difficulties (*R* ^2^ = 0.25). In this model, diagnosed child mental disorder was significantly associated with child difficulties, *B* = 4.66, *p* = 0.025.

**TABLE 4:** Results of linear regression models.

Predictor	Outcome	*N*	*B*	SE	95% CI^a^ [LL, UL]	*β*	*p*	*R* ^*2*^
BSI GSI	SDQ TD^a^	57	5.83	1.58	[2.67, 9.00]	0.44	< 0.001	0.25
BSI GSI	PRFQ PM	62	1.03	0.20	[0.63, 1.42]	0.56	< 0.001	0.31
PRFQ PM	SDQ TD^a^	57	2.90	0.86	[1.19, 4.62]	0.41	0.001	0.23

Abbreviations: *β*, standardised coefficient beta; *B*, unstandardized regression coefficient; BSI GSI, Brief Symptom Inventory Global Severity Index (range 0–53); CI, confidence interval; LL, lower limit; PRFQ PM, Parental Reflective Functioning Questionnaire Pre‐Mentalizing (range 1–7); SDQ TD, Strength and Difficulties Questionnaire Total Difficulties (range 0–40); SE, standard error; UL, upper limit.

^a^ Child mental disorder was entered as a covariate.

### 3.4 Parental Psychiatric Symptom Severity and Pre‐Mentalizing

In support of our second hypothesis, a simple linear regression model showed that higher parental psychiatric symptom severity was significantly related to higher levels of pre‐mentalizing (Table 4). Parental psychiatric symptom severity explained 31% of the variance in pre‐mentalizing (*R* ^2^ = 0.31).

### 3.5 Pre‐Mentalizing and Child Emotional and Behavioural Difficulties

In accordance with our third hypothesis, a final multiple linear regression model showed that higher pre‐mentalizing (predictor) was associated with more child emotional and behavioural difficulties (outcome), when child mental disorder was entered as a covariate (Table 4). This model explained 23% of the variance in child difficulties (*R* ^2^ = 0.23). Child mental disorder was a significant covariate in the model, *B* = 4.84, *p* = 0.021.

### 3.6 Mediation Analysis

**FIGURE 2:**
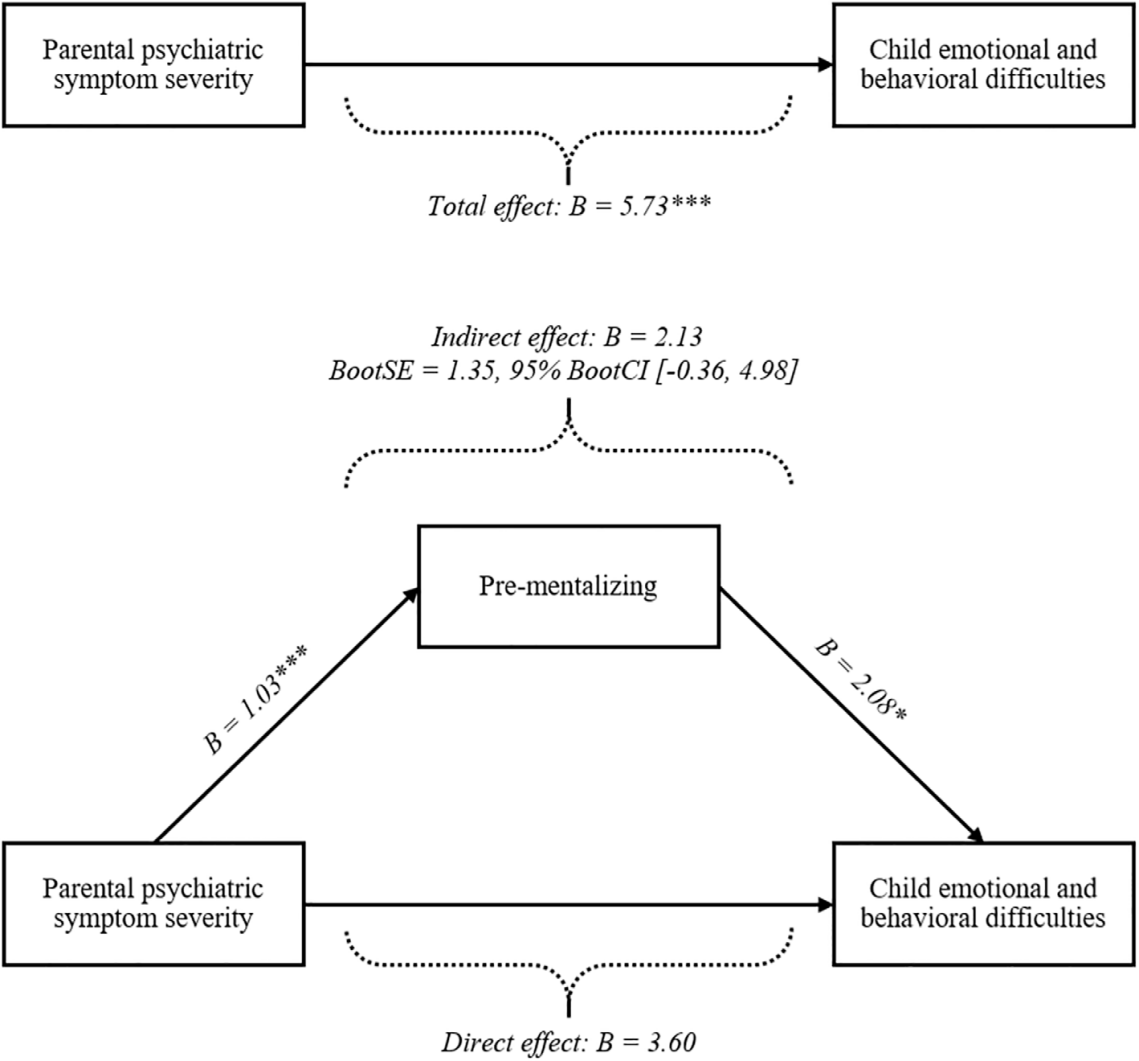
Illustration of the total, indirect and direct effects with unstandardized coefficients. *N* = 56. * = *p <* 0.05, ** *= p* *<* 0.01, *** *= p <* 0.001. Child mental disorder was entered as a covariate in the mediation analysis.

The mediation analysis revealed a significant positive relationship between parental psychiatric symptom severity and child emotional and behavioural difficulties (i.e., total effect), *B* = 5.73, SE = 1.58, *t* = 3.63, *p* < 0.001. When pre‐mentalizing was entered as a mediator, the effect of parental psychiatric symptom severity on child difficulties was no longer significant (i.e., direct effect), *B* = 3.60, SE = 1.82, *t* = 1.97, *p* = 0.054. There was a significant positive association between parental psychiatric symptom severity and pre‐mentalizing, *B* = 1.03, SE = 0.22, *t* = 4.74, *p* < 0.001. Even when controlling for parental psychiatric symptom severity, there was a significant link between pre‐mentalizing and child difficulties, *B* = 2.08, SE = 0.97, *t* = 2.14, *p* = 0.037. However, the indirect effect of parental psychiatric symptom severity on child emotional and behavioural difficulties through pre‐mentalizing was not significant, *B* = 2.13, BootSE = 1.35, 95% BootCI [−0.36, 4.98]. Thus, pre‐mentalizing was not found to be a significant mediator in discordance with hypothesis four. The coefficients for the paths in the mediation model are presented in Figure 2.

## 4 Discussion

The aim of this study was to explore the links between parental psychiatric symptom severity, PRF, and child emotional and behavioural difficulties, and to test these in a mediation model in a clinical outpatient sample of parents with a diagnosed mental disorder. Parental psychiatric symptom severity predicted child emotional and behavioural difficulties as well as pre‐mentalizing, which in turn were associated with child emotional and behavioural difficulties. However, PRF was not found to mediate the relationship between parental psychiatric symptom severity and child emotional and behavioural difficulties.

Our first finding that parental symptom severity predicted child emotional and behavioural difficulties adds to a large body of literature linking parental psychopathology with early signs of psychopathology in their children (Connell and Goodman 2002; Dean et al. 2018; Watkeys et al. 2022). Research investigating the role of parental psychiatric symptom severity in the transmission of risk has mainly focused on depressive symptoms (e.g., Ivanova et al. 2022). Global measures of psychiatric symptom severity have primarily been linked to child emotional and behavioural difficulties in community samples (e.g., Oliver and Midouhas 2023; Suveg et al. 2011), and therefore our study contributes to the field by replicating this finding in a clinical sample of parents with a diagnosed mental disorder.

Our second finding indicates that parents with higher levels of psychiatric symptoms were more likely to adopt a non‐mentalizing approach to their children, consistent with the theoretical expectation that psychological distress leads to heightened risk of disruptions in mentalizing (Luyten, Nijssens, et al. 2017). Our finding is in line with a meta‐analysis finding depressive symptoms to be associated with pre‐mentalizing (Georg et al. 2023). However, we find a somewhat more robust relationship, which could be due to the clinical status of our sample, as the meta‐analysis primarily included community‐based studies. Other than depressive symptoms, pre‐mentalizing has been linked to the severity of anxiety (De Palma et al. 2023; Krink and Ramsauer 2021), paranoid ideation (Paris et al. 2015), trauma (Paris et al. 2022), and borderline symptoms (Kamza et al. 2024). Our finding in a sample of parents with various mental disorders adds to an emerging body of research which altogether provides preliminary support for PRF being a transdiagnostic construct. Furthermore, few studies have examined pre‐mentalizing in relation to global measures of severity spanning multiple symptom domains. While Wendelboe et al. (2021) found global severity to predict pre‐mentalizing in a sample of mothers scoring above cut‐off for symptoms of postpartum depression, other studies have not detected this association in parents with personality disorders (Hestbaek et al. 2024) and substance use disorders (Paris et al. 2015). Our finding thus extends prior research that has yielded mixed results.

Our third finding that higher levels of pre‐mentalizing predicted increased child emotional and behavioural difficulties is congruent with the theoretical assumption that pre‐mentalizing is characterised by deficient or distorted interpretations about the child's state of mind, resulting in caregiving that is not sufficiently sensitive or emotionally responsive to the child, in line with findings of previous studies (Luyten, Mayes, et al. 2017; Krink et al. 2018). By not having their mental states understood and adequately mirrored back to them by their caregiver, the child's capacity for emotional regulation and self‐development may not be sufficiently scaffolded, which is supported by studies linking pre‐mentalizing to insecure attachment (Luyten, Mayes, et al. 2017) and impaired socio‐emotional development in offspring (Gordo et al. 2020; Madsen et al. 2023; Nijssens et al. 2020). Furthermore, our finding is consistent with recent studies finding associations between pre‐mentalizing and child emotional and behavioural difficulties in community samples (e.g., Charpentier Mora et al. 2022; Dieleman et al. 2020; Khoshroo and Mousavi 2022). By replicating this finding in a clinical sample of parents, our study emphasises the importance of addressing pre‐mentalizing in parents with mental disorders to promote adaptive parenting and child development in their high‐risk children. Considering our sample's average pre‐mentalizing score being in the low end of the subscale (*M* = 2.13), our study indicates that even slightly elevated levels of pre‐mentalizing may be associated with increased risk of mental health problems in offspring, supporting the notion of pre‐mentalizing as an indicator of impaired PRF with strong predictive value (Luyten, Nijssens, et al. 2017; Madsen et al. 2023).

Contrary to our fourth hypothesis, pre‐mentalizing did not mediate the association between parental psychiatric symptom severity and child emotional and behavioural difficulties. A possible explanation for why we did not detect a mediational effect has to do with our modest sample size, which was limited to 56 participants in the mediation analysis. As previously stated, the existing research on such a mediation model is scarce, with studies examining comparable models in large community samples yielding inconsistent findings (Keleynikov et al. 2024; Raouna et al. 2025). Keleynikov et al. (2024) found that pre‐mentalizing mediated the relationship between parental symptoms of depression and child emotion regulation in a sample of parents (*N* = 732) of children aged 3–7 years. Raouna et al. (2025) found pre‐mentalizing to mediate the link between parental hypomanic traits and child socio‐emotional development but detected no mediation when assessing depressive symptoms in a sample of parents (*N* = 1788) of children under the age of 2. Due to the well‐established risk for psychopathology among children of parents with a mental disorder (Leijdesdorff et al. 2017; Van Santvoort et al. 2015), it is plausible that the relationship between parental psychiatric symptom severity and child difficulties is stronger in our sample compared to community‐based samples, creating even more difficult conditions for pre‐mentalizing to act as a mediator. Although the indirect effect (i.e., mediation) did not reach statistical significance in the current study, the direct effect of parental psychiatric symptom severity on child difficulties became insignificant when pre‐mentalizing was included in the model. This, in conjunction with the confidence interval for the indirect effect, indicates that the mediating role of pre‐mentalizing may be trending towards significance. Thus, the results of our mediation analysis must be regarded as tentative, and future research could benefit from testing the model in larger samples of parents with a mental disorder.

### 4.1 Suggestions for Future Research and Clinical Practice

Although we did not find support for the mediational role of PRF, our results still suggest that impaired PRF is closely related to the severity of psychiatric symptoms among parents with a mental disorder as well as early signs of mental health problems in their children. These preliminary findings point to the importance of further investigating the role of PRF in families where a parent suffers from a mental disorder. In doing so, future research should seek to apply more advanced and nuanced measures of parental mentalizing and child emotional and behavioural difficulties to overcome the limitations of parental self‐report.

To this date, there exist no cut‐off scores on the PRFQ (Anis et al. 2020), making it difficult to interpret what levels are indicative of adaptive and maladaptive PRF. A task for future research could be to develop such thresholds to promote the scale's applicability in making assessments in clinical practice. Furthermore, as with other studies, the internal consistency of the pre‐mentalizing subscale of the PRFQ was relatively low, necessitating further evaluation. Future research could benefit from developing implicit measures of PRF, utilising experimental and/or observational approaches to examine PRF in high‐arousal situations (Law et al. 2021; Luyten et al. 2019). Efforts in this regard were recently demonstrated by Malcorps et al. (2025) using a baby‐simulator paradigm.

It remains important for future research to assess child well‐being when studying parents with a mental disorder. Ratings of child difficulties differ depending on the informant, and future studies may benefit from using multi‐informant methods to gain more nuanced and accurate descriptions of the child's problems (Van Der Ende et al. 2012). The SDQ has been shown to be a more sensitive predictor of psychiatric disorders in children when assessed by multiple compared to single‐informant reports (Goodman et al. 2003). Besides reducing bias from parental self‐report, obtaining multiple accounts of a child's strengths and difficulties could provide insight into how the child copes in various contexts.

Although we did not find support for the mediating role of PRF, the findings of our study support existing literature demonstrating an association between psychiatric symptom severity in parents and child emotional and behavioural difficulties in samples of parents with a mental disorder (Ivanova et al. 2022; Sweeney and Wilson 2023). In terms of clinical practice, this highlights the importance of preventative interventions to promote the mental well‐being of these high‐risk children. A number of different parenting interventions have been developed for this purpose and have proven to be effective, with meta‐analysis of randomised controlled trials demonstrating a 47% reduction in offspring risk of developing a mental disorder (Lannes et al. 2021).

Furthermore, our study suggests that PRF appears to be diminished among those parents with the most severe psychiatric symptoms. As there is evidence that PRF can indeed be improved by parenting programmes (Lo and Wong 2022), there may be clinical potential in paying attention to PRF among this group of parents.

### 4.2 Strengths and Limitations

This study has several strengths. Whilst there is a growing body of research regarding PRF and its relation to parental psychiatric symptom severity and child difficulties, studies have largely been carried out in community samples, highlighted as an issue by multiple scholars (e.g., Georg et al. 2023; Nijssens et al. 2020). With our clinical sample of parents recruited from a psychiatric outpatient clinic, we thus fill an important gap in the literature as the children of these parents are at particularly high risk of adverse outcomes, including emotional and behavioural problems. Although theory has long proposed a strong link between PRF and child development, research on this topic has been limited and has rarely included child outcome measures (Camoirano 2017). Therefore, the current study's inclusion of these measures is a major advantage. Another strength is our use of broad‐band measures, which allowed us to capture a great variance in the manifestations of both parental psychiatric symptoms and child emotional and behavioural difficulties, accounting for comorbidity, which was high for the parents in our sample. Furthermore, this approach accounts for the non‐concordant transmission of psychopathology demonstrated by prior research (Dean et al. 2018; McLaughlin et al. 2012; Uher et al. 2023).

However, there are several limitations of this study. A primary limitation is the cross‐sectional design, which precludes causal inference and prevents clear determination of the temporal sequencing required for mediation. Nevertheless, our model is grounded in theoretical assumptions about variable ordering and is supported by longitudinal research linking parental psychiatric symptoms with child difficulties (Flouri et al. 2019) and parental mentalizing (Bigelow et al. 2018), as well as parental mentalizing with child difficulties (Salo et al. 2021). Parenting interventions that improve PRF find support for reduction in child difficulties (Byrne et al. 2020; Lavender et al. 2023), consistent with the proposed ordering of PRF in this study. Additionally, the use of a high‐risk clinical parent sample supports the proposed model. Longitudinal research is needed to test temporal pathways. Furthermore, given the weak to moderate effects in the existing literature and the large sample sizes required to detect such in mediation analysis (Fritz and MacKinnon 2007), it is likely that our mediation analysis was underpowered and at risk of type II error (Field 2013).

Our sample included parents with diverse psychiatric diagnoses and children ranging from 0 to 17 years, reflecting considerable heterogeneity with consequences for the precision and generalizability of our findings. The heterogeneity of the sample likely increased variability, potentially reducing the precision of effect estimates. Furthermore, the wide age range and focus on a global index of parental symptoms restricted our ability to examine more nuanced associations, such as those specific to particular child age groups or parental symptom domains.

Another limitation of this study pertains to all variables being self‐reported. The fact that all scales were reported by the same parent introduces the risk of shared method variance, potentially inflating associations (Podsakoff et al. 2003). Social desirability bias and wanting to present themselves as a successful parent could lead respondents to overrate their own abilities to mentalize their child and to underrate their child's difficulties, as could the fear of child protection services or loss of custody (Maybery and Reupert 2009). However, the clinical status of the sample could also influence the results in the opposite direction. As parents' ratings of their child's difficulties have been found to be related to their levels of symptoms (Treutler and Epkins 2003), SDQ scores in our study might be affected by a negative rating bias. Furthermore, self‐reports of PRF and child difficulties may be subject to self‐assessment bias particularly among parents with low PRF, since parents with difficulties in mentalizing struggle with reporting the mental states of themselves and others (Fonagy et al. 2016). Interview‐ and observation‐based measures, such as the Mind‐Mindedness observation and the Parent Development Interview, overcome this issue of self‐report (Schiborr et al. 2013). However, these measures have been criticised for lacking sensitivity in identifying distorted reflective functioning (Luyten, Mayes, et al. 2017; Slade and Sleed 2024). Like the PRFQ, these measures are limited in capturing how parental mentalizing may switch off under high‐arousal situations (Law et al. 2021).

Furthermore, some challenges pertaining to our use of the PRFQ should be noted. Our use of the scale among a broader age range of children than the scale was originally developed for could have consequences for the interpretations of our results, potentially contributing to the relatively low internal consistency of the pre‐mentalizing subscale. However, similar findings have been reported in other studies, indicating that this issue is not confined to the broader age range of our sample (Krink et al. 2018; Kungl et al. 2024; Wendelboe et al. 2021).

## 5 Conclusion

This study aimed to investigate the associations between parental psychiatric symptom severity, PRF, and child emotional and behavioural difficulties, and to explore the potential mediating role of PRF among a sample of parents with diagnosed mental disorders. The results showed that parental psychiatric symptom severity was positively associated with higher levels of child emotional and behavioural difficulties as well as pre‐mentalizing, which in turn was predictive of child emotional and behavioural difficulties. Our finding that parents with more severe psychiatric symptoms to a higher degree report early signs of offspring mental health problems and non‐mentalizing assumptions about their child's state of mind underlines the importance of considering symptom severity among parents with a mental disorder. Contrary to expected, we did not find pre‐mentalizing to mediate the association between parental symptom severity and child emotional and behavioural difficulties. However, as our findings are preliminary, replication is warranted in efforts to further study the role of pre‐mentalizing in the transmission of psychopathology from parent to child.

## Funding

The authors have nothing to report.

## Conflicts of Interest

The authors declare no conflicts of interest.

## Data Availability

Research data are not shared.
